# Advances in understanding and managing atopic dermatitis

**DOI:** 10.12688/f1000research.6972.1

**Published:** 2015-11-19

**Authors:** Michael Barton, Robert Sidbury

**Affiliations:** 1 University of Washington School of Medicine, Seattle, WA, 98195, USA; 2 Department of Pediatric Dermatology, Seattle Children’s Hospital, Seattle, WA, 98105, USA

**Keywords:** dermatitis, atopic, immune

## Abstract

Atopic dermatitis is a chronic, pruritic skin disease characterized by an improperly functioning skin barrier and immune dysregulation. We review proposed atopic dermatitis pathomechanisms, emphasizing how these impact current perspectives on natural history, role of allergic sensitization, and future therapeutic targets.

## Introduction

Atopic dermatitis (AD) is a common chronic, pruritic skin disease characterized by an improperly functioning skin barrier and immune dysregulation. Part of the “atopic triad”, it often precedes asthma and seasonal allergies, affecting up to 20% of children and 3% of adults in the United States ^1, 2^. The age-old question remains: is AD primarily a skin barrier defect or a dysfunctional immune system? This dichotomy is made explicit in various clinical scenarios. For example, patients with primary immunodeficiency diseases like hyper-IgE (Job’s) syndrome, some with IgE levels of more than 30,000 IU/ml, often have terrible AD, but so do some hypogammaglobulinemia or severe combined immunodeficiency patients who may have little to no IgE at all. Additionally, 80% of patients with AD have a positive atopic history with increased serum IgE levels or immediate skin test reactivity or both, but an identical eczema phenotype is seen in the other 20% of patients with AD, those with so-called intrinsic AD, who have no atopic tendency at all. Furthermore, certain areas of the skin have notoriously been affected (e.g., flexural surfaces of the elbow/knee), whereas other areas appear to be spared (i.e., axilla), which would seem incongruent with constitutive immune dysregulation. This construct extends to the therapeutic realm as immunosuppressive agents like cyclosporin A have demonstrated efficacy, but so too have emollients and barrier repair “devices” that putatively do not affect the immune system.

So which is the chicken and which the egg? Does barrier dysfunction beget immune dysregulation or vice versa? Improved understanding of the genetic and molecular bases of AD will likely answer this question and, as will be detailed later, lead to better therapies. It may well have broader implications vis-à-vis the “atopic march”: the orderly procession of infantile eczema, to childhood asthma, to adult hay fever that occurs in some patients. A variety of studies, including early application of emollients to high-risk infants, have suggested that not only can eczema be interrupted but other co-morbidities can potentially be prevented ^3^.

## Skin barrier abnormalities: the “outside-in” theory

The skin barrier is composed of multiple elements working synchronously to protect the host from various external insults. The physical barrier is composed of the outermost layer of the epidermis, or stratum corneum, and deeper tight junctions. A broad range of proteins, including but not limited to S100 family proteins and filaggrin byproducts, contribute to the skin’s physical integrity. The normal host microbiome plays a pivotal role in the protection of skin from pathogens as well as the determination of immune responses. Immunologically, several barrier components have been shown to be altered in patients with AD. This was elegantly demonstrated by Ong *et al.*, who showed that patients with AD, notorious for being colonized and infected by *Staphylococcus aureus*, had deficient levels of antimicrobial peptides in biopsy specimens relative to patients with psoriasis, an inflammatory dermatosis with many similarities, except the tendency toward infection ^4^. Both genetic and acquired causes of epithelial skin barrier dysfunction have been identified. Recent advances have focused on the primary importance of this abnormal barrier as a trigger for eczematous inflammation. This “outside-in” construct purports that leakage in the barrier allows the penetration of various allergens, irritants, and microbes into the skin of patients with AD, thereby leading to a cascade of inflammatory events.

Although several barrier defects have previously been examined, recent literature seems to be directed toward filaggrin as a major culprit in the development and severity of AD ^5^. Initially demonstrated to have reduced expression in patients with ichthyosis vulgaris, an inherited disorder of keratinization, and a minor diagnostic criterion for AD, filaggrin’s direct association with AD has been elucidated over the last decade ^6^. Filaggrin is the monomeric breakdown product of a polyprotein encoded on the *FLG* gene. It serves stratum corneum structure and function as well as inhibits transepidermal water loss. Loss-of-function mutations in the *FLG* gene are a major risk factor for AD, and even variations in gene size have been associated with an increased AD risk ^7^. Within the AD population, these mutations lead to early onset, more severe course, and higher prevalences of asthma, food allergy, and microbial infection ^8– 10^.

Regardless of *FLG* gene status, filaggrin deficiency alone appears to play an important role in the pathogenesis of AD ^11^. Deficiencies in filaggrin-processing enzymes such as caspase-14 have been identified ^12^. Environmental stressors such as low ambient humidity, sunburn, skin irritants, and psychological stress have been proposed to reduce filaggrin levels in the epidermis. Additionally, inflammation itself can downregulate filaggrin expression, revealing the interdependent relationship between skin barrier and immune dysregulation in AD ^13^. Interestingly, current therapies such as topical steroids and calcineurin inhibitors have been shown to restore filaggrin levels back to normal, and new therapies are being studied to directly target the modulation of *FLG* expression ^14, 15^.

Similar reductions of other structural and tight junction proteins such as loricrin, involucrin, and claudins contribute to the abnormal barrier in AD. Emerging evidence suggests that collectively these defects may contribute to systemic allergic sensitization. A recent study revealed an exposure-response relationship between peanut protein levels found in household dust and peanut skin prick test (SPT) sensitization in patients with AD ^16^. The authors conclude that increased allergen exposure through an impaired skin barrier in inflamed skin may be responsible for sensitization and subsequent allergy. Results from the LEAP (Learning Early about Peanut Allergy) trial, a study designed to assess the development of peanut allergy in infants at high risk for the allergy (which included infants with severe eczema or egg allergy or both), suggest that early introduction of peanuts into an infant’s diet may promote oral tolerance and prevent peanut allergy ^17^. Upon being randomly assigned to either consume or avoid peanuts, 640 infants between the ages of 4 and 11 months were followed until they reached 5 years of age. Among the infants who initially had negative SPTs, the prevalence of peanut allergy was significantly higher among the avoidance group (13.7%) compared with the consumption group (1.9%). Moreover, those infants who initially demonstrated SPT positivity had peanut allergy prevalences of 35.3% in the avoidance group and 10.6% in the consumption group. Collectively, these data support the notion of skin sensitization and oral tolerance among patients with AD, which may impact the approach to high-risk pediatric patients in the future. Early food consumption appears to reduce the risk of developing specific systemic allergy sensitization, but further information is needed to determine whether improved control of skin barrier defects during infancy will have the same effect. These studies raise many additional questions. Should all patients be exposed to peanut early? All eczema patients? Do these data extend to other allergenic foods? If so, when to introduce? In what form? How much? These answers require further study, and the National Institutes of Health has assembled an expert panel to incorporate these important new findings into their previously published food allergy guidelines ^18^.

## Immune system dysfunction: the “inside-out” theory

Immune dysregulation is critical to the pathogenesis of AD. Several existing therapies target T cell-mediated inflammation. The molecular signature of AD inflammation varies by chronicity and phase of the disease, but T _H_2, T _H_22, and T _H_17 cells predominate, resulting in characteristic inflammatory mediators, including interleukin-4 (IL-4) and IL-13. These cells and their inflammatory cytokines correspondingly have an effect on the epidermal barrier, including suppression of skin cell differentiation, hyperplasia, and apoptosis. Specific cytokines appear to downregulate terminal differentiation genes responsible for protective barrier proteins, including filaggrin, loricrin, and involucrin. Immune activation has a complex and intertwined relationship with the skin barrier defects of patients with AD, and continuing to identify immunologic pathways will have profound effects on understanding and managing AD.

## Advances in treatment

One of the most exciting candidate drugs in this arena is dupilumab. Previously shown to be effective in patients with asthma and elevated eosinophil levels, dupilumab is an injectable, fully human, monoclonal antibody that blocks the cellular messenger signals IL-4 and IL-13 ^19^. In a randomized, double-blind, placebo-controlled trial, Beck *et al*. showed marked improvement in EASI 50 (50% reduction in Eczema Area and Severity Index) score compared with placebo (85% versus 35%, *P* < 0.001) ^20^. Additionally, patients receiving dupilumab reported a significant decrease in symptomatic itching, and when used in combination, dupilumab served as a topical steroid-sparing agent.

Apremilast is an oral phosphodiesterase 4 (PDE-4) inhibitor currently used in the treatment of psoriasis. In an uncontrolled, open-label pilot study of 16 adults with moderate to severe AD receiving either 20 or 30 mg twice daily, clinical responses at 6 months were similar to those seen with systemic immunosuppressants, including cyclosporine, mycophenolate, and methotrexate, based on EASI scores and quality-of-life indices ^21, 22^. The ability to improve inflammation, itch, and quality of life while avoiding end-organ damage seen with alternative systemic agents is an appealing characteristic of this drug. Although these results provide a strong foundation to build upon, additional controlled studies with greater sample sizes are necessary to determine efficacy, dosage, and safety.

Several topical PDE-4 inhibitors are also being studied but the furthest along is AN2728 ^23^. In one phase 2 trial, 86 adolescents with mild to moderate AD receiving twice-daily AN2728 treatment revealed a 71% improvement from baseline in their Atopic Dermatitis Severity Index (ADSI) score and total or partial clearance in 66% of the skin lesions ^24^. On the basis of such encouraging phase 2 data, two phase 3 trials with 750 patients with mild to moderate AD have been conducted. AN2728, now named crisaborole, was shown to be superior to vehicle at day 29 in the percentage of patients found clear or almost clear by global assessment: 32.8% versus 25.4% ( *P* < 0.04) in one trial and 31.4% versus 18% ( *P* < 0.001) in the other. Pooled analysis of adverse effects showed that only application site pain was seen at rates above vehicle (4.4% versus 1.2%). Crisaborole appears to be a safe and effective non-steroidal alternative for mild to moderate eczema. Time will tell where it may fit into the therapeutic armamentarium and what current agent it may function most like, but these trial results suggest efficacy akin to pimecrolimus.

Omalizumab is a humanized monoclonal antibody that selectively binds to IgE antibodies. It has been licensed for the treatment of severe allergic asthma and chronic urticaria. Its effectiveness treating severe and treatment-resistant AD has been controversial, and mixed results have derived from several small case studies and uncontrolled trials ^25, 26^. One randomized, double-blind, placebo-controlled study of eight patients found comparable improvements in AD between omalizumab and placebo ^27^. A 2014 study assessed the efficacy of omalizumab in patients with and without *FLG* mutations ^28^. None of the seven *FLG* mutation carriers responded, whereas the eight patient responders had no *FLG* mutations, suggesting that patients with a primary skin barrier defect may not respond to anti-IgE therapy. The potential benefit of omalizumab for patients with AD is uncertain at this time and will depend upon the results of additional randomized control trials.

Several lines of investigation have suggested a role for vitamin D in AD pathogenesis ^29– 31^. There is good evidence to support AD improvement with vitamin D supplementation ^32– 35^. An initial study conducted in 2008 revealed that 1,000 IU of vitamin D improved AD in four of the five children studied, compared with only one of the six children in the control group. Subsequent studies have reproduced similar results with larger sample sizes. Despite these encouraging trial results, attempts to correlate AD severity and vitamin D levels have led to mixed results ^36^. Important questions remain, including optimal dosing and target serum levels.

Thymic stromal lymphopoietin (TSLP) is another intriguing target for directed therapeutic interventions. TSLP is an epithelial cell-derived cytokine responsible for triggering the differentiation of naïve T cells into T _H_2 cells and has been shown to have an association with AD and other allergic diseases ^37– 39^. Variations within the TSLP-encoding gene have also been associated with a decreased risk of persistent AD and susceptibility to eczema herpeticum ^40^. The first human anti-TSLP drug to be tested, AMG 157, has exhibited promising results in the reduction of allergen-induced asthmatic responses ^41^. Although the effectiveness of AMG 157 for management of AD has not been studied directly, it is reasonable to predict, given the known relationship between AD and T _H_2 cells, that the drug may show similar results in the skin.

It would seem prudent to target therapy directly toward pruritus, for a condition known as “the itch that rashes”; however, there is currently no treatment available to single out this neural pathway. CT327 and tradipitant are two medications in phase 2 development aiming to address this aspect of therapy by antagonizing tropomyosin-receptor kinase A (trkA) and neurokinin 1 receptors (NK-1Rs), respectively. A 2010 study of 15 AD patients with moderate pruritus revealed noticeable symptomatic improvements within 8 days with topical CT327 ^42^. Itch symptoms improved by up to 59%, and no serious side effects were experienced; CT327 has also shown promising results for the treatment of chronic pruritus in patients with psoriasis ^43^. Similar outcomes are being measured by focusing on NK-1R, which has a high affinity for substance P, a neurotransmitter believed to influence a variety of pathophysiological processes, including itch. Higher blood concentrations of oral tradipitant have been correlated with greater improvements in itch scores compared with control groups ^44^.

## Conclusions

The future for patients with AD has never looked so promising. Advances in understanding the genetic basis of AD and its associated barrier defects are dovetailing with a better grasp of the molecular basis of eczematous inflammation. The result will hopefully lead to improved therapies in the short term and possible prevention of both AD and atopic co-morbidities down the road.

## Abbreviations

AD, atopic dermatitis; ADSI, Atopic Dermatitis Severity Index; EASI, eczema area and severity index; IL, interleukin; NK-1R, neurokinin 1 receptor; PDE-4, phosphodiesterase 4; TSLP, thymic stromal lymphopoietin.
